# Infection with SARS-CoV-2 during pregnancy and risk of stillbirth: a Scandinavian registry study

**DOI:** 10.1136/bmjph-2023-000314

**Published:** 2023-10-25

**Authors:** Maria Christine Magnus, Anne Kristina Örtqvist, Stine Kjaer Urhoj, Anna Aabakke, Laust Hvas Mortensen, Håkon Gjessing, Anne-Marie Nybo Andersen, Olof Stephansson, Siri Eldevik Håberg

**Affiliations:** 1 Centre for Fertility and Health, Norwegian Institute of Public Health, Oslo, Norway; 2 Department of Medicine, Karolinska Institute, Stockholm, Sweden; 3 Department of Obstetrics and Gynaecology, Visby County Hospital, Visby, Sweden; 4 Department of Public Health, University of Copenhagen, Kobenhavn, Denmark; 5 Statistics Denmark, Copenhagen, Denmark; 6 Department of Obstetrics and Gynecology, Copenhagen University Hospital, Kobenhavn, Denmark; 7 Department of Clinical Medicine, University of Copenhagen, Kobenhavn, Denmark; 8 Department of Global Public Health and Primary Care, University of Bergen, Bergen, Norway; 9 Department of Women's Health, Karolinska University Hospital, Stockholm, Sweden

**Keywords:** COVID-19, risk assessment, public health

## Abstract

**Background:**

A few studies indicate that women infected with SARS-CoV-2 during pregnancy might have an increased risk of stillbirth. Our aim was to investigate the risk of stillbirth according to infection with SARS-CoV-2 during pregnancy also taking the variant into account.

**Methods:**

We conducted a register-based study using the Swedish, Danish and Norwegian birth registries. A total of 389 949 births (1013 stillbirths) after 22 completed gestational weeks between 1 May 2020 and end of follow-up (27 January 2022 for Sweden and Norway; 31 December 2021 for Denmark). We estimated the risk of stillbirth following SARS-CoV-2 infection after 22 completed gestational weeks using Cox regression for each country, and combined the results using a random-effects meta-analysis.

**Results:**

SARS-CoV-2 infection after 22 completed gestational weeks was associated with an increased risk of stillbirth (adjusted HR 2.40; 95% CI 1.22 to 4.71). The risk was highest during the first weeks following infection, with an adjusted HR of 5.48 (95% CI 3.11 to 9.63) during the first 2 weeks, 4.38 (95% CI 2.41 to 7.98) during the first 4 weeks, and 3.71 (95% CI 1.81 to 7.59) during the first 6 weeks. Furthermore, the risk was greatest among women infected during the Delta-dominated period (adjusted HR 8.23; 95% CI 3.65 to 18.59), and more modest among women infected during the Index (adjusted HR 3.66; 95% CI 1.89 to 7.06) and Alpha (adjusted HR 2.73; 95% CI 1.13 to 6.59) dominated periods.

**Conclusions:**

We found an increased risk of stillbirth among women who were infected with SARS-CoV-2 after 22 gestational weeks, with the greatest risk during the Delta-dominated period.

**WHAT IS ALREADY KNOWN ON THIS TOPIC**

**WHAT THIS STUDY ADDS**

- Using information on 389 949 births in Sweden, Denmark and Norway, we found a clear increased risk of stillbirth among women who had tested positive for SARS-CoV-2 from 22 gestational weeks and onwards.
- We further found evidence of some variation in the risk of stillbirth according to SARS-CoV-2 variants, with the greatest risk among women infected during the Delta-dominated period.

**HOW THIS STUDY MIGHT AFFECT RESEARCH, PRACTICE OR POLICY**

- The findings from this study support the existing recommendation for vaccination of pregnant women.
- We also highlight the importance of continued evaluation of differences in the risk of pregnancy complications with new any variants of the SARS-CoV-2 virus.

## Introduction

 The impact of SARS-CoV-2 infection on the risk of adverse pregnancy outcomes remains to be fully understood. Pregnant women are at higher risk of severe COVID-19 disease, which increases the risk of pregnancy complications.^1^,^3^ Women infected with SARS-CoV-2 during pregnancy have a greater risk of preterm birth, and higher risk of giving birth to infants with low Apgar score, poor intrauterine growth and infants admitted to neonatal intensive care.^1 4 5^ Several studies have attempted to assess the risk of stillbirth according to infection with SARS-CoV-2; however, most studies only included between 2 and 30 exposed cases of stillbirth (only 2 studies had more than 10 exposed cases), yielding mixed results.^6^,^11^

The largest study of SARS-CoV-2 and stillbirth included 1 249 634 births between March 2020 and September 2021 registered in the US Premier Healthcare Database Special COVID-19 Release (PHD-SR), with a total of 21 653 births to women with COVID-19, and 273 exposed cases of stillbirth. They found an increased risk of stillbirth with infection (adjusted relative risk of 1.90; 95% CI 1.69 to 2.15).^12^ Emerging evidence further suggests differences in the risk of pregnancy complications according to SARS-CoV-2 virus variants.^13 14^ Evidence regarding differences in the risk of stillbirth according to variants remains sparse.^12 15 16^

In previous studies, we have showed that there has been no notable difference in the overall rate of stillbirth in the Nordic countries.^17^ Furthermore, findings from Sweden and Norway indicated no increased risk of stillbirth with vaccination against SARS-CoV-2. In the current study, we build on these studies by investigating whether SARS-CoV-2 infection after 22 gestational weeks was associated with risk of stillbirth, with combined data on 389 949 births between 2020 and 2022 in Sweden, Denmark and Norway. We also evaluated the risk of stillbirth among women infected with SARS-CoV-2 at times dominated by different viral variants, as studies have indicated a greater risk of pregnancy complications following infection with the Delta variant.^12 16^

## Methods

### Study population

We studied live and stillbirths after 22 completed gestational weeks in Sweden, Denmark and Norway between 1 March 2020 and end of follow-up (27 January 2022 for Sweden and Norway; 31 December 2021 for Denmark). Births were identified through the Swedish Pregnancy Register,^18^ the Danish National Patient Register (registrations of International Classification of Disease version 10 codes Z38, O80–84 and P95),^19^ and the Medical Birth Registry of Norway.^20 21^ The small number of late induced abortions conducted after 22 completed gestational weeks were excluded (n=110 for Denmark; n=23 for Norway; information not available for Sweden). The Danish and Norwegian data included all births nationally, while the Swedish data included 94% of all births in Sweden (in 18 of 21 Swedish regions). We only included singletons and only the first registered birth to each woman during the study period. To avoid oversampling of preterm pregnancies towards end of the study period, we excluded pregnancies without the possibility to reach 42 completed weeks by the end of follow-up. We obtained information on maternal socioeconomic measures, infections with SARS-CoV-2, and vaccination against SARS-CoV-2 from national databases using unique national identification numbers.

### Stillbirth

Stillbirth was defined as a fetal death after 22 completed gestational weeks. Gestational age was estimated based on ultrasound for the majority of births (more than 90% in all countries), and on date of last menstrual period when ultrasound estimates were missing. Information on the method of pregnancy dating is available from all registries. We used 22 completed gestational weeks and not 20 gestational weeks because only births occurring after 22 completed gestational weeks were available in the Swedish Pregnancy Register.

### SARS-CoV-2 infection

The exposure of interest was a positive test for SARS-CoV-2 after 22 completed gestational weeks up until the day before delivery. Our hypothesis is that infection may increase the risk of fetal death. Thus, infection before gestational week 22 might increase the risk of fetal death prior to 22 completed weeks (miscarriage), which could affect our results when only looking at fetal deaths after 22 gestational weeks (stillbirths). We did not exclude women with infection prior to 22 gestational weeks from the analysis, but adjusted for this as a covariate. In addition, we conducted a sensitivity analysis excluding pregnancies to women infected during pregnancy prior to 22 completed gestational weeks. The beginning of pregnancy was estimated based on the date of birth minus the gestational age in days. Information on laboratory-confirmed PCR positive tests for SARS-CoV-2 was obtained from mandatory reports to SmiNet at the Public Health Agency for Sweden,^22^ and from the Norwegian Surveillance System for Communicable Diseases for Norway,^23^ while information on both PCR and antigen positive tests was available from the Microbiology Database at the State Serum Institute for Denmark.^24 25^ In Denmark, 10% of the positive tests were antigen tests, while 90% were PCR. The testing strategies in the three countries have varied over time. We stopped the follow-up of this study in January 2022, as all three Nordic countries changed their testing strategies around this time, and no longer recommended testing of all symptomatic individuals. More details about the testing strategies across the countries are available in online supplemental file.

### Covariates

We obtained information on maternal age at the beginning of pregnancy (continuous), parity (0, 1, 2 or more), educational level (9 years or less, 10–12 years, more than 12 years), household income in tertiles based on the national distribution (first, second and third tertile), living with a partner (yes or no), region of birth (Scandinavia, Other European countries, Middle East/Africa, other/unknown), smoking in pregnancy (yes or no), pre or early-pregnancy body mass index (continuous), pre-existing chronic condition (yes or no) prior to pregnancy and vaccination against SARS-CoV-2 (none, before pregnancy, during pregnancy). A general recommendation for vaccination of all pregnant women was issued in May 2021, in Sweden, August 2021, in Norway and July 2021, in Denmark. Pre-existing chronic conditions prior to pregnancy included hypertension, chronic kidney disease, asthma, cardiovascular disease, thrombosis and diabetes. We also obtained information on pre-eclampsia, gestational diabetes, placental abruption, uterine rupture and shoulder dystocia from the birth and patient registries. These pregnancy complications were not adjusted for in the multivariable model because they may represent potential mediating pathways.

### Statistical analysis

We used Cox regression analysis to evaluate the HR of stillbirth according to SARS-CoV-2 infection. The time axis was gestational age in days, and follow-up started at 22 completed gestational weeks (gestational day 154). The end of follow-up in the Cox model was the gestational day of birth. Infection with SARS-CoV-2 was entered as a time-varying exposure, so women could contribute both unexposed and exposed follow-up time. First, we evaluated the risk according to infection any time after 22 completed gestational weeks. To evaluate whether the risk was different during the first weeks after infection, we estimated the risk of stillbirth in separate analyses using 2, 4 and 6 weeks exposure windows following infection with SARS-CoV-2, and women were considered as unexposed after the end of the specific risk window. In multivariable analyses, we adjusted for age, parity, educational level, household income, living with a partner, region of birth and a time-varying variable of vaccination against SARS-CoV-2. We also adjusted for infection with SARS-CoV-2 during pregnancy prior to 22 completed gestational weeks in the main model. We subsequently adjusted for smoking during pregnancy and early or prepregnancy body mass index in births with this information available (87% of all births). The data from each country were analysed separately, and the results were subsequently meta-analysed using a random-effects model, with heterogeneity estimated using the I^2^ statistic. Country specific results are not shown due to national privacy regulations for presenting small numbers.

In secondary analyses, we evaluated differences in the risk of stillbirth according to a positive test during time periods dominated by different variants of SARS-CoV-2; (Index (prior to 1 February 2021), Alpha (between 1 February 2021 and 30 June 2021) or Delta (between 1 July 2021 and 31 December 2021)). The dates for cut offs for the variants were based on the major circulating variant at the time,^26^,^28^ which was similar across the three countries. Finally, we conducted sensitivity analyses excluding women who were infected within 4 weeks prior to 22 completed gestational weeks. In addition, to compare women with the same possibility of being infected throughout pregnancy, we performed a sensitivity analysis restricted to pregnancies with an estimated start after 1 March 2020. These sensitivity analyses were only done for the 4-week risk window analysis. We further conducted a sensitivity analysis defining those who tested positive the last 3 days of pregnancy as unexposed. We also conducted a sensitivity analysis excluding women infected during pregnancy prior to 22 completed gestational weeks.

Analyses were conducted using STATA V.17 (Statacorp) and R V.4.2.1.

### Patient and public involvement

No patients were involved in setting the research question or the outcome measures, nor were they involved in developing plans for recruitment, design or implementation of the study. No patients were asked to advise on interpretation or writing up of results. There are no plans to disseminate the results of the research to study participants or the relevant patient community.

## Results

**Figure 1:**
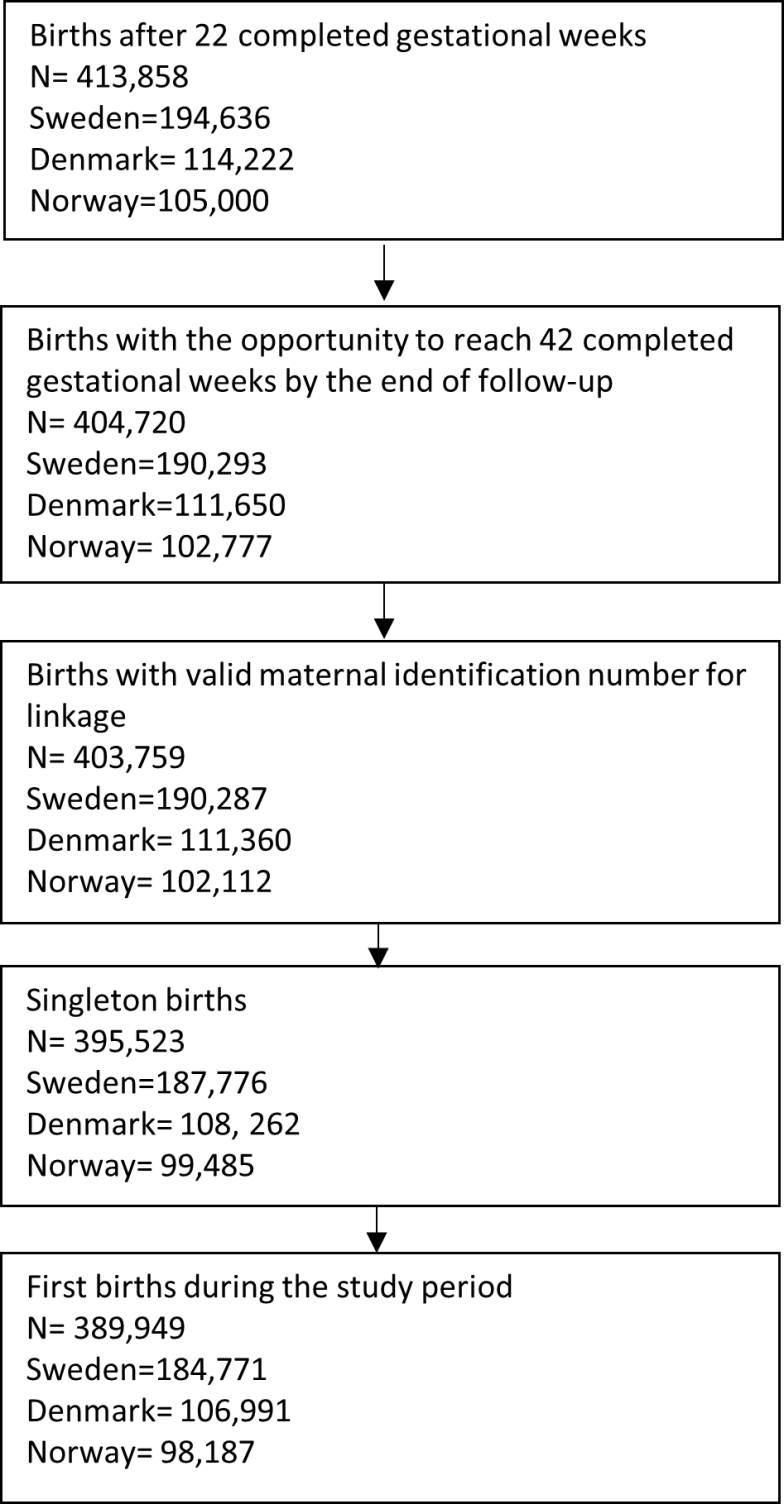
Illustration of study population.

Across the three countries, there were 389 949 births during the study period (figure 1), with 184 771 in Sweden, 106 991 in Denmark and 98 187 in Norway. Among these births, 1013 (3 per 1000) ended in a stillbirth (3 per 1000 in Sweden, 3 per 1000 in Denmark and 2 per 1000 in Norway). There were 8855 births (2.3%) to women with a positive test for SARS-CoV-2 in pregnancy after gestational week 22 (3.2% in Sweden, 1.7% in Denmark and 0.9% in Norway). Among women with positive tests after 22 gestational weeks, a total of 31 women had a subsequent stillbirth. Women who tested positive for SARS-CoV-2 during pregnancy had a slightly higher parity, lower educational level, lower income, were more likely to be from the middle East/Africa, and had a slightly higher prepregnancy body mass index, compared with women who did not test positive during pregnancy (table 1). These characteristics were similar across the three countries. Women who had underlying chronic conditions were more likely to have tested positive for SARS-CoV-2 in Denmark, less likely to have tested positive in Norway, while there was no apparent difference in Sweden (table 1).

**Table 1:** Maternal characteristics according to SARS-CoV-2 infection after 22 completed gestational weeks

Country	Sweden		Denmark		Norway	
Background characteristics	Without infection(n=178 675)	With infection(n=6096)	Without infection(n=105 161)	With infection(n=1830)	Without infection(n=97 258)	With infection(n=929)
Age at start of pregnancy, mean (SD)	30.7 (4.8)	30.6 (4.8)	30.8 (4.7)	30.6 (4.8)	30.7 (4.7)	30.7 (4.8)
Parity, No. (%)						
0	77 550 (43.4)	2442 (40.1)	50 490 (48.0)	866 (47.3)	41 898 (43.1)	333 (35.8)
1	66 197 (37.1)	2240 (36.8)	39 330 (37.4)	653 (35.7)	36 280 (37.3)	344 (37.0)
≥2	34 928 (19.6)	1414 (23.2)	15 341 (14.6)	311 (17.0)	19 080 (19.6)	252 (27.1)
Missing						
Educational level, No. (%)						
≤9 years	16 030 (9.0)	595 (9.8)	10 581 (10.1)	221 (12.1)	12 762 (13.1)	196 (21.1)
10–12 years	67 714 (37.9)	2460 (40.4)	28 464 (27.1)	535 (29.2)	18 722 (19.3)	172 (18.5)
>12 years	86 071 (48.2)	2749 (45.1)	64 537 (61.4)	1034 (56.5)	56 778 (58.4)	392 (42.2)
Missing	8860 (5.0)	292 (4.8)	1579 (1.5)	40 (2.2)	8996 (9.3)	169 (18.2)
Household income, No. (%)						
First tertile	58 544 (32.8)	1924 (31.6)	33 830 (32.2)	685 (37.4)	30 806 (31.7)	375 (40.4)
Second tertile	58 397 (32.7)	2071 (34.0)	34 347 (32.7)	520 (28.4)	30 905 (31.8)	276 (29.7)
Third tertile	58 469 (32.7)	1998 (32.8)	34 320 (32.6)	583 (31.9)	30 979 (31.9)	202 (21.7)
Missing	3265 (1.8)	103 (1.7)	2664 (2.5)	42 (2.3)	4568 (4.7)	76 (8.2)
Living with partner, No. (%)						
Yes	161 458 (90.4)	5590 (91.7)	88 787 (84.4)	1500 (82.0)	91 763 (94.4)	859 (92.5)
No	12 978 (7.3)	398 (6.5)	15 905 (15.1)	321 (17.5)	3870 (4.0)	54 (5.8)
Missing	4239 (2.4)	108 (1.8)	469 (0.5)	9 (0.5)	1625 (1.7)	16 (1.7)
Region of origin, No. (%)*						
Scandinavia	127 173 (71.2)	4056 (66.5)	87 743 (83.4)	1366 (74.6)	72 282 (74.3)	446 (48.0)
Middle East/ Africa	27 933 (15.6)	1232 (20.2)	4860 (4.6)	214 (11.7)	6625 (6.8)	217 (23.4)
Other European countries	14 611 (8.2)	555 (9.1)	7530 (7.2)	162 (8.9)	10 836 (11.1)	160 (17.2)
Other/unknown	8958 (5.0)	253 (4.2)	5028 (4.8)	88 (4.8)	7515 (7.7)	106 (11.4)
Any chronic disease, No. (%)†	19 617 (11.0)	670 (11.0)	13 045 (12.4)	244 (13.3)	8584 (8.8)	55 (5.9)
Smoking in pregnancy, No. (%)						
No	166 668 (93.3)	5715 (93.8)	93 962 (89.4)	1635 (89.3)	82 398 (84.7)	763 (82.1)
Yes	5938 (3.3)	172 (2.8)	7497 (7.1)	101 (5.5)	4950 (5.1)	51 (5.5)
Missing	6069 (3.4)	209 (3.4)	3702 (3.5)	94 (5.1)	9910 (10.2)	115 (12.4)
Prepregnancy body mass index median (IQR)	24 (22–28)	25 (22–28)	24 (21–27)	24 (22–27)	24 (21–27)	24 (22–28)
SARS-CoV-2 vaccination status, No. (%)						
None	158 888 (88.9)	5871 (96.3)	96 510 (91.8)	1722 (94.1)	88 895 (91.4)	829 (89.2)
Before pregnancy	280 (0.2)	<5 (0.1)	210 (0.2)	12 (0.7)	228 (0.2)	11 (1.2)
During pregnancy	19 507 (10.9)	222 (3.6)	8.441 (8.0)	96 (5.3)	8135 (8.4)	89 (9.6)
Infected with SARS-CoV-2 prior to 22 completed gestational weeks	5868 (3.3)	0	1642 (1.6)	0	605 (0.6)	0
Gestational age						
22–27 weeks	445 (0.3)	19 (0.3)	275 (0.3)	<5 (0.2)	248 (0.3)	<5 (0.2)
28–31 weeks	722 (0.4)	32 (0.5)	432 (0.4)	6 (0.3)	392 (0.4)	<5 (0.4)
32–36 weeks	6813 (3.8)	278 (4.6)	4117 (3.9)	56 (3.1)	3705 (3.8)	26 (2.8)
37–41 weeks	166 954 (93.4)	5652 (92.7)	98 000 (93.2)	1713 (93.6)	89 215 (91.7)	864 (93.0)
42 weeks or more	3741 (2.1)	115 (1.9)	2337 (2.2)	52 (2.8)	3698 (3.8)	33 (3.6)
Spontaneous preterm birth						
No	173 395 (97.0)	5935 (97.4)	101 636 (96.7)	1788 (97.7)	94 707 (97.4)	915 (98.5)
Yes	5280 (3.0)	161 (2.6)	3525 (3.4)	42 (2.3)	2551 (2.6)	14 (1.5)
Pre-eclampsia						
No	172 444 (96.5)	5884 (96.5)	101 502 (96.5)	1763 (96.3)	94 697 (97.4)	911 (98.1)
Yes	6231 (3.5)	212 (3.5)	3635 (3.5)	67 (3.7)	2561 (2.6)	18 (1.9)
Missing			24 (0.02)	0 (0)		
Small-for-gestational age						
No	160 864 (90.0)	5487 (90.0)	94 579 (98.9)	1624 (88.7)	89 149 (91.7)	828 (89.1)
Yes	15 088 (8.4)	530 (8.7)	10 013 (9.5)	189 (10.3)	8096 (8.3)	101 (10.9)
Missing	2723 (1.5)	79 (1.3)	569 (0.5)	17 (0.9)	13 (0.01)	0 (0)
Gestational diabetes						
No	169 488 (94.9)	5708 (93.6)	99 642 (94.8)	1716 (93.8)	91 417 (94.0)	855 (92.0)
Yes	9187 (5.1)	388 (6.4)	5495 (5.2)	114 (6.2)	5841 (6.0)	74 (8.0)
Missing			24 (0.02)	0 (0)		

* The other category included North America, South America, Latin America, Asia, Australia and New Zeeland.

† Chronic conditions included hypertension, chronic kidney disease, asthma, cardiovascular disease, thrombosis, and diabetes. These are all conditions associated with both the risk of COVID-19 and adverse pregnancy complications.

The median gestational age for the positive test for SARS-CoV-2 after 22 completed gestational weeks pregnancy was 214 days (IQR 184–245) in Sweden, 217 days (IQR 185–251) in Denmark and 228 days (IQR 193–257) in Norway. There were a total of 31 exposed cases of stillbirth across the three countries. All of the 31 exposed cases of stillbirth occurred among unvaccinated women. These exposed cases were infected between 31 March 2020 and 28 October 2021. Most exposed stillbirths (19 of 31) occurred within 3 weeks after a positive test. We observed an increased risk of stillbirth among women who were infected with SARS-CoV-2 after 22 completed gestational weeks, with an incidence rate of 6 per 100 000 exposed follow-up days and 2 per 100 000 unexposed follow-up days, with a corresponding adjusted HR of 2.40 (95% CI 1.22 to 4.71), and evidence of heterogeneity between countries (I^2^ 68%; p=0.04; table 2). We also found that only four of the exposed cases of stillbirth were to women who had been admitted to the ICU during pregnancy for COVID-19. The risk of stillbirth was highest during the first weeks after infection with SARS-CoV-2, with an adjusted HR of 5.48 (95% CI 3.11 to 9.63) during the 2 weeks following infection, 4.38 (95% CI 2.41 to 7.98) during the 4 weeks following infection, and 3.71 (95% CI 1.81 to 7.59) during the 6 weeks following infection (table 2). Further adjustment for body mass index and smoking did not influence the results (online supplemental etable 1). A total of 6 out of 31 exposed stillbirths were the result of spontaneous preterm birth, while <5 were exposed to pre-eclampsia, and 16 were small-for-gestational age. Only eight exposed cases of stillbirth occurred more than 6 weeks after infection. They occurred between gestational day 174 and 282. None of the exposed cases of stillbirth had any registration of uterine rupture, placental abruption or shoulder dystocia. None of the exposed cases of stillbirth were to women who had a history of a previous stillbirth.

**Table 2:** Risk of stillbirth with maternal SARS-CoV-2 infection after 22 completed gestational weeks

Exposure window	SARS-CoV-2 infection	Follow-up time in days	No of events	Unadjusted		Adjusted*	
				HR (95% CI)	I^2^ heterogeneity statistic†	HR (95% CI)	I^2^ heterogeneity statistic†
2 weeks	Unexposed	43 647 189	999	Ref		Ref	
	Exposed	119 109	14	6.24 (3.13 to 12.45)	38%, p=0.20	5.48 (3.11 to 9.63)	10%, p=0.33
4 weeks	Unexposed	43 530 188	993	Ref		Ref	
	Exposed	236 110	20	4.72 (2.36 to 9.45)	56%, p=0.10	4.38 (2.41 to 7.98)	41%, p=0.19
6 weeks	Unexposed	43 413 334	990	Ref		Ref	
	Exposed	352 964	23	3.86 (1.74 to 8.57)	72%, p=0.03	3.71 (1.81 to 7.59)	64%, p=0.06
Any time after 22 gestational weeks	Unexposed	43 228 574	982	Ref		Ref	
	Exposed	537 724	31	2.84 (1.24 to 6.47)	79%, p=0.01	2.40 (1.22 to 4.71)	68%, p=0.04

* Adjusted for maternal age at start of pregnancy, parity, education, income, living with a partner, region of birth, underlying chronic conditions and vaccination against SARS-CoV-2.

† The I2 heterogeneity statistic and corresponding p- value for differences in the estimates across the three countries.

The risk of stillbirth was highest in women infected during the Delta-dominated period, with incidence rates of 16 per 100,000, while it was 5 per 100 000 during the Index variant period, and 4 per 100 000 during the Alpha variant period (table 3). In the 4 weeks following infection with SARS-CoV-2 during the Delta-dominated period, the adjusted HR was 8.23 (95% CI 3.65 to 18.59), while the adjusted HR was 3.66 (95% CI 1.89 to 7.06) for the Index variant, and 2.73 (95% CI 1.13 to 6.59) for the Alpha period (table 3). There was substantial less heterogeneity in the variant-specific estimates between countries than in the main analysis (table 3).

**Table 3:** Risk of stillbirth after maternal infection with different variants of SARS-CoV-2 after 22 completed gestational weeks

Exposure window	SARS-CoV-2 infection	Follow-up time in days	No of stillbirths	Unadjusted		Adjusted*	
				HR (95% CI)	I^2^ heterogeneity statistic†	HR (95% CI)	I^2^ heterogeneity statistic†
4 weeks risk window	Unexposed	33 579 188	993	Ref		Ref	
	Index	116 807	9	3.66 (1.90 to 7.05)	0%, p=0.71	3.66 (1.89 to 7.06)	0%, p=0.74
	Alpha	92 066	5	2.75 (1.14 to 6.61)	0%, p=0.53	2.73 (1.13 to 6.59)	0%, p=0.65
	Delta	27 133	6	9.95 (4.45 to 22.24)	0%, p=0.69	8.23 (3.65 to 18.59)	0%, p=0.80
Any time after 22 weeks	Unexposed	43 228 574	982	Ref		Ref	
	Index	269 778	12	1.60 (0.90 to 2.84)	0%, p=0.82	1.51 (0.85 to 2.68)	0%, p=0.91
	Alpha	211 203	10	2.56 (0.86 to 7.61)	64%, p=0.06	2.16 (0.85 to 5.47)	51%, p=0.13
	Delta	56 639	9	6.15 (3.18 to 11.88)	0%, p=0.70	4.88 (2.49 to 9.56)	0%, p=0.88

* Adjusted for maternal age at start of pregnancy, parity, education, income, living with a partner, region of birth, underlying chronic conditions and vaccination against SARS-CoV-2.

† The I2 heterogeneity statistic and corresponding p- value for differences in the estimates across the three countries.

When we excluded women who were infected within 4 weeks prior to 22 completed gestational weeks, or restricted the analysis to pregnancies starting after 1 March 2020, we observed similar results for the 4-week risk window (online supplemental etables 2 and 3). We also observed a similar increased risk of stillbirth according to infection with SARS-CoV-2 when defining those who tested positive the last 3 days of pregnancy as unexposed (online supplemental etable 4). Excluding women infected with SARS-CoV-2 during pregnancy prior to 22 completed gestational weeks also did not change the results (online supplemental etable 5).

## Discussion

In this Scandinavian registry-based study, we observed an increased risk of stillbirth among women infected with SARS-CoV-2 after 22 completed gestational weeks, with the highest risk in the first weeks following a positive SARS-CoV-2 test. The risk was highest among women who tested positive for SARS-CoV-2 when Delta was the major circulating variant.

### Strengths and weaknesses of this study

Important strengths of this study include the population-covering and prospective nature of the registry data, the inclusion of data from three countries, the evaluation of the risk according to dominant variants of SARS-CoV-2, and our ability to adjust for several potential confounding factors. Our study also has limitations. We did not have any information on causes and classifications of stillbirths in the birth registries, including clinical information from autopsies or placental histopathological analysis. Furthermore, no information on SARS-CoV-2 infection of the fetus was available. We were also unable to look at the risk of stillbirth according to the gestational week of infection with SARS-CoV-2 in more detail due to the small number of exposed cases. There were also differences in test strategies and infection rates between countries and during the study period. From Sweden and Norway, we only had information on PCR-positive tests, while from Denmark, information on rapid antigen tests were also available. During the study period, individuals in Denmark who tested positive on a rapid antigen test were recommended to take a confirmatory PCR-test, and 90% of the positive tests from Denmark in this study were PCR, ensuring comparability between the countries. Among women with positive tests, there are likely some asymptomatic cases, as pregnant women exposed to infected individuals were recommended to take a PCR test.^29^ Also, women with early signs of fetal loss may have been more prone to testing which could inflate risk estimates. However, in the sensitivity analysis, where we defined pregnancies to women who tested positive the last 3 days of pregnancy as unexposed, results were similar. The risk of stillbirth was highest with infection during the Delta period in all three countries, which support that increased testing in threatening stillbirths is less likely to cause the associations. We were unable to distinguish the risk of stillbirths prior to the onset of labour and stillbirths arising intrapartum according to infection with SARS-CoV-2 due to small numbers. We also did not have any information on treatment for SARS-CoV-2, and we could, therefore, not evaluate how it might have impacted the subsequent risk of stillbirth. Finally, we had limited ability to evaluate the proportional hazards assumption due to the small number of exposed cases of stillbirth.

### Comparison with previous studies

Our findings support previous studies indicating that SARS-CoV-2 infection increases the risk of stillbirth. The largest study to date from the US PHD-SR, found a twofold increased risk of stillbirth among women with a diagnosis of COVID-19 around the time of delivery.^12^ A study of 78 centres included in the Spanish Obstetric Emergency Group, including 1347 SARS-CoV-2 PCR-positive pregnant women registered between 26 February 2020 and 5 November 2020 on admission for delivery, and a concurrent sample of PCR-negative mothers, also indicated a greater proportion of stillbirth among women who were positive for SARS-CoV-2 compared with those who were negative (0.7% vs 0.2%, p=0.02).^6^ Similarly, a population-based study from England which included 342 080 delivering women, of which 3527 were registered in the birth record as being positive for SARS-CoV-2 at the time of delivery, the adjusted relative risk of stillbirth was 2.21 (95% CI 1.58 to 3.11).^8^

To study differences with viral variants, we used information on the major underlying circulating variant at the time when the woman tested positive. We observed the greatest increased risk of stillbirth among women who tested positive in the Delta dominated period. As our follow-up ended in January 2022, the number of stillbirths was too low to study the risk of stillbirth according to infection with the Omicron variant. Some other studies support that the Delta variant could have a greater impact on the risk of stillbirth compared with other variants. The US (PHD-SR) study found that the risk of stillbirth was greater in women infected with SARS-CoV-2 during the Delta period (adjusted RR 4.04; 95% CI 3.28 to 4.97), and lower in the pre-Delta period (adjusted RR 1.47; 95% CI 1.27 to 1.71).^12^ A population-based study from Scotland with 9817 women who tested positive for SARS-CoV-2 during pregnancy, also reported a lower rate of stillbirth in the Omicron-dominant period (4.3 per 1000 births) than in the Delta-dominant period (20.3 per 1000 births).^16^

We observed some differences in the associations across countries as evidenced by the heterogeneity statistics. There was substantially less heterogeneity when examining infection according to the major circulating variant at the time the women were infected with SARS-CoV-2. Statistical heterogeneity may be present due to small numbers in each of the countries, whereas clinical heterogeneity may be caused by national differences in the intensity of the pandemic itself or differences in testing strategies that led to differences in the proportion of positive cases detected across countries. We used random-effects meta-analysis to account for this heterogeneity in the country-combined estimates.

Some mechanistic evidence support that the SARS-CoV-2 virus can influence the risk of stillbirth. A histological analysis of the placenta from 15 women with severe COVID-19 who delivered in the third trimester indicated abnormal vessels and evidence of intervillous thrombi.^30^ Another study from Sweden of 14 placentas also supported massive perivillous fibrinoid deposition, in addition to intervillositis and tromphoblast necrosis.^31^ Notably, vascular malperfusion is commonly found in the placenta of stillbirths after COVID-19 infection.^32^ There is also evidence suggesting that the Delta variant of the SARS-CoV-2 could increase the risk of stillbirth. Histological evidence of one intrauterine fetal demise in an unvaccinated woman with mild symptoms of SARS-CoV-2, suggested that the excessive infiltration of immune cells and cytokines in the placenta due to the Delta variant caused severe placental inflammation and damage, which likely resulted in placental abruption and the demise of the fetus.^33^ However, the impact of the SARS-CoV-2 on the placenta still remains to be fully understood, as the existing studies are based on very small sample sizes.

## Conclusion

In this Scandinavian registry-based study, infection with SARS-CoV-2 was associated with an increased risk of stillbirth, with the greatest risk among women exposed to the Delta variant, although the small number of exposed cases yielded uncertain estimates for the individual variants. We also did not have information available on the fetus’ infection status. However, our findings highlight the need for further understanding of differences in risk of pregnancy complications according to SARS-CoV-2 variants. The tendency for an increased risk of stillbirth among women infected with SARS-CoV-2 during pregnancy highlights the importance of vaccination of pregnant women which was recommended across most countries.

## supplementary material

10.1136/bmjph-2023-000314 online supplemental file 1

## Data Availability

Data may be obtained from a third party and are not publicly available.
