# Supplementary material for: Infection with SARS-CoV-2 during pregnancy and risk of stillbirth: a Scandinavian registry study
Source: BMJ Public Health. 2023 Oct 25;1(1):e000314. doi: 10.1136/bmjph-2023-000314 (PMC11812723; doi:10.1136/bmjph-2023-000314)
Supplement: online supplemental file 1 [file bmjph-1-1-s001.pdf]

Supplementary Appendix

This supplementary material has been provided by the authors to give readers additional information about their work.

Supplement to: Infection with SARS-CoV-2 during pregnancy and risk of stillbirth: a Scandinavian registry study

Maria C. Magnus, Anne K. Örtqvist, Stine Kjaer Urhoj, Anna J. M. Aabakke, Laust H. Mortensen, Håkon K. Gjessing, Anne-Marie Nybo Andersen, Olof Stephansson, Siri E. Håberg

Table of contents

Data sources and linkages in Sweden…………………………………………………………………………p. 3

Data sources and linkages in Norway…………………………………………………………………………p. 4

Data sources and linkages in Denmark………………………………………………………………………..p. 5

eTable 1 Risk of stillbirth according to maternal history of SARS-CoV-2 infection after 22 completed gestational weeks exploring additional adjustment for body-mass index and smoking……p. 6

eTable 2 Risk of stillbirth according to maternal history of SARS-CoV-2 infection after 22 completed gestational weeks looking at the 4 weeks risk window excluding those who tested positive the last 4 weeks before 22 completed gestational weeks…………………………………………p. 7

eTable 3 Risk of stillbirth according to maternal history of SARS-CoV-2 infection after 22 completed gestational weeks restricting to pregnancies starting March 1st 2020 looking at the 4 weeks risk window…………………………………………………………………………………………………..p. 8

eTable 4 Risk of stillbirth with maternal SARS-CoV-2 infection after 22 completed gestational weeks sensitivity analysis defining those testing positive the last 3 days of pregnancy as unexposed………p. 9

eTable 5 Risk of stillbirth with maternal SARS-CoV-2 infection after 22 completed gestational weeks sensitivity analysis excluding those who had tested positive any time prior to 22 competed gestational weeks……………………………………………………………………………………………p. 10

References………p.11

Data sources and linkages in Sweden

The Swedish Pregnancy register (SPR)
Data in this study was provided through the Swedish Pregnancy Register. This quality register was initiated in 2013, and includes 94% of all births in Sweden (18 of 21 regions). Demographical, reproductive and maternal health care data, starting at the first visit to the antenatal care clinic around the ninth gestational week, are transferred from electronic medical records within 24 hours from a reported birth. The register includes information on birth outcomes in addition to maternal background characteristics, health during pregnancy, pregnancy and neonatal outcomes.

The Swedish Neonatal Quality Register (SNQ)
Th SNQ includes all infants born alive in Sweden who were admitted for neonatal care within 27 days after birth. For this study we used information on neonatal care admission.

The national vaccination register in Sweden
As of 1 January 2013, healthcare providers must report all vaccinations administered within the Swedish vaccination programs to the Swedish vaccination register, held by the Public Health Agency of Sweden. The register includes type and date of all Covid-19 vaccinations.

Swedish Register for Communicable Diseases (SmiNet)
In Sweden, SARS-CoV-2 was included in the Swedish Communicable Disease Act on 1st February, 2020, making all laboratory-confirmed polymerase chain reaction (PCR) cases of SARS-CoV-2 mandatory to report within 24 hours to SmiNet at the Public Health Agency of Sweden.[1] Negative tests results are unfortunately not available on a national level. A non-universal population testing strategy was implemented including outpatient testing and contact tracing, starting in June 2020 and ongoing to January 2022. This type of testing mostly included symptomatic individuals, but it could also include individuals tested before and after travels, or after contact with other test-positive individuals and individuals subject to workplace testing (e.g. healthcare workers).[2] In 23 of the 39 delivery hospitals covered by the Swedish Pregnancy Register, universal testing of all women admitted for labor or pregnancy in-patient care, independent of their current and previous medical history and COVID-19 symptoms or not, were performed.[3] From mid-January 2022, testing decreased due to new recommendations. Individuals are no longer required to get tested, but to stay at home if one has symptoms.[4]

Data sources and linkages in Norway

The Emergency Preparedness Register for Covid-19
Data in this study were provided through the Emergency preparedness register for Covid-19 (Beredt C19) administered by the Norwegian Institute of Public Health, according to the Health Preparedness Act §2-4. This registry was established in 2020 to provide authorities with up to date information on prevalence, causal relationships, and consequences of the Covid19 epidemic in Norway. Beredt C19 includes information already collected in the healthcare service, national health registries and administrative registers with information about the Norwegian population. The data subjects' right is safeguarded as they can contact the data controller for all different sources included in Beredt C19 in the usual way. Through Beredt C19 we used data from the following sources:

The Norwegian Immunisation Register (SYSVAK)
SYSVAK is a register of vaccines in the Norwegian vaccination program, with mandatory registrations of all Covid-19 vaccinations (dates and type).

Norwegian Surveillance System for Communicable Diseases (MSIS)
The Norwegian Surveillance System for Communicable Diseases (MSIS) contains mandatory reporting of selected infectious diseases.[5] Reporting of all PCR tests for SARS-CoV-2 to this registry is mandatory, and it contains both the date of testing and test results. Both negative and positive tests results from all PCR tests are reported to this registry. It does not contain any information on antigen tests for SARS-CoV-2, and in particular, it does not contain any information on home-based tests which became increasingly common during the pandemic. Notably, everyone who tested positive on an antigen test were recommended to get a confirmatory PCR test. In Norway, testing strategies and tests capacities varied greatly across the country during the pandemic. Primarily symptomatic individuals were tested, and Norway had a PCR test capacity of ~40000/day by the summer of 2020.[6] Other reasons for being tested included direct contact with confirmed test-positive individuals, workplace testing (such as for example health-care workers), in addition to testing in connection with travel restriction. There was at no point a universal testing strategy for pregnant women admitted to hospital (including for delivery). From the end of January, 2022, individuals were no longer recommended to get tested for SARS-CoV-2 even though they had symptoms, but were rather asked to stay home until they were symptoms free.

Statistics Norway (SSB)
Administrative data is mandatorily reported to Statistics Norway. We used information from this database on household income in 2018, type of education and years of education completed by 2019.

The Medical Birth Registry of Norway (MBRN)
The Norwegian national birth registry includes information on all pregnancies ending in gestational week 12 or later. The registry includes information on birth outcomes in addition to maternal background characteristics, health during pregnancy, pregnancy outcomes and neonatal health.

Data sources and linkages for Denmark

The Danish National Patient Register (DNPR)

The Danish National Patient Registry was established in 1977. The register contains individual-level longitudinal registration of all contacts to Danish hospitals (inpatient admissions and non-primary outpatient admissions), including dates and codes for diagnosis, births, medical surgery etc. Diagnosis codes are coded according to the International Classification of Diseases system. The validity and the completeness of the register vary according to clinical specialties; for the clinical specialty of obstetrics and gynecology the validity is generally considered to be high. In this study, the DNPR was used to identify all women giving birth after 22 gestational weeks, as the Danish Medical Birth Register has not been updated since 2018 (as per November 2022).

Danish Microbiology Database (MiBa)

The Danish Microbiology Database (MiBa) was established in 2010 and contains complete data on all microbiological samples performed in Denmark from general practices, test centres and hospitals.[7] Besides SARS-CoV-2 test results from PCR tests, MiBa also includes rapid antigen tests from December 2020 performed at official test centres and other places reporting the test results to the database. Both SARS-CoV-2 PCR test and rapid antigen tests are free of charge in Denmark (not including home test kits), easily accessible and has been widely used. In case of a positive rapid antigen test result, the person was recommended to have a PCR test to confirm the result. The nationwide testing strategies and test capacities varied during the study period and rapidly evolved from March/April 2020, where mostly symptomatic people were tested and with a PCR-test capacity of around 2000 tests/day. During May 2020 an intensive contact trace strategy was put in place where all persons who had been in contact with a person with SARS-CoV-2 infection was isolated and tested. Universal testing of all pregnant women admitted to hospital (including for delivery) was also implemented in May 2020.[8] The test capacity increased to around 20000 PCR-tests/day in June 2020. During 2020 the test capacity increased to around 90000 PCR-tests/day in December. The test capacity topped in March 2021 with around 170.000 PCR-tests/day. From 21 July 2021, COVID vaccination was recommended for pregnant women in their second and third trimester. For a detailed description of the COVID-19 timeline from January 2020-December 2022 see covid-19-tidslinje-lang-for-2020-2022-version-2---december-2022.pdf (ssi.dk) (in Danish).

Vaccination Register

The Danish Vaccination Register (DDV) covers all vaccines administered to Danish patients and the reporting to the register has been mandatory since 2015 (e.g. all Covid-19 vaccinations are registered with date and type). COVID vaccination was recommended for pregnant women in their second and third trimester from July 21, 2021.

Statistics Denmark

Information about educational levels, income, living with a partner and region of birth was obtained from population registers at Statistics Denmark.

eTable 1 Risk of stillbirth according to maternal history of SARS-CoV-2 infection after 22 completed gestational weeks exploring additional adjustment for body-mass index and smoking

**Exposure window**	**SARS-CoV-2 infection during pregnancy**	**Follow-up time in days**	**Number of events**	**Unadjusted**		**Adjusted ***		**Adjusted †**	
				HR (95% CI)	I^2 heterogeneity statistic ‡	HR (95% CI)	I^2 heterogeneity statistic ‡	HR (95% CI)	I^2 heterogeneity statistic ‡
**2 weeks**	Unexposed	41624181	926	Ref		Ref		Ref	
	Exposed	113778	12	5.10 (2.88 to 9.01)	0%, 0.51	4.83 (2.73 to 8.55)	0%, 0.51	4.82 (2.72 to 8.54)	0%, 0.49
**4 weeks**	Unexposed	41512365	920	Ref		Ref		Ref	
	Exposed	225594	18	4.03 (2.40 to 6.78)	14%, 0.31	3.92 (2.34 to 6.57)	12%, 0.32	3.93 (2.31 to 6.69)	16%, 0.31
**6 weeks**	Unexposed	41400559	917	Ref		Ref		Ref	
	Exposed	337400	21	3.44 (1.81 to 6.55)	49%, 0.14	3.38 (1.80 to 6.37)	46%, 0.16	3.39 (1.78 to 6.47)	48%, 0.14
**Any time after 22 weeks**	Unexposed	41223437	909	Ref		Ref		Ref	
	Exposed	514522	29	2.61 (1.28 to 5.33)	68%, 0.04	2.27 (1.21 to 4.27)	58%, 0.09	2.28 (1.20 to 4.33)	60%, 0.08

* Adjusted for maternal age at start of pregnancy, parity, education, income, living with a partner, region of birth, underlying chronic conditions and vaccination against SARS-CoV-2.

† Adjusted for maternal age at start of pregnancy, parity, education, income, living with a partner, region of birth, underlying chronic conditions, vaccination against SARS-CoV-2, smoking and body-mass index.

‡ The I^2 heterogeneity statistic and corresponding p-value for differences in the estimates across the three countries.

eTable 2 Risk of stillbirth according to maternal history of SARS-CoV-2 infection after 22 completed gestational weeks looking at the 4 weeks risk window excluding those who tested positive the last 4 weeks before 22 completed gestational weeks

SARS-CoV-2 infection during pregnancy	Follow-up time in days	Number of events	Unadjusted HR (95% CI)	Adjusted HR (95% CI) *
**Unexposed**	43325286	992	Ref	Ref
**Exposed**	209499	19	5.36 (2.39 to 12.01)	4.55 (2.35 to 8.80)
**I^2 heterogeneity statistic †**			66%, p-value 0.05	50%, p-value 0.14

* Adjusted for maternal age at start of pregnancy, parity, education, income, living with a partner, region of birth, underlying chronic conditions and vaccination against SARS-CoV-2.

† The I^2 heterogeneity statistic and corresponding p-value for differences in the estimates across the three countries.

eTable 3 Risk of stillbirth according to maternal history of SARS-CoV-2 infection after 22 completed gestational weeks restricting to pregnancies starting March 1st 2020 looking at the 4 weeks risk window

SARS-CoV-2 infection during pregnancy	Follow-up time in days	Number of events	Unadjusted HR (95% CI)	Adjusted HR (95% CI) *
**Unexposed**	27627755	628	Ref	Ref
**Exposed**	214432	18	4.80 (2.25 to 10.22)	4.40 (2.26 to 8.55)
**I^2 heterogeneity statistic †**			60%, p-value 0.08	48%, p-value 0.15

* Adjusted for maternal age at start of pregnancy, parity, education, income, living with a partner, region of birth, underlying chronic conditions and vaccination against SARS-CoV-2.

† The I^2 heterogeneity statistic and corresponding p-value for differences in the estimates across the three countries.

eTable 4 Risk of stillbirth with maternal SARS-CoV-2 infection after 22 completed gestational weeks sensitivity analysis defining those testing positive the last 3 days of pregnancy as unexposed

**Exposure window**	**SARS-CoV-2 infection**	**Follow-up time in days**	**Number of events**	**Unadjusted**		**Adjusted** *	
				HR (95% CI)	I^2 heterogeneity statistic †	HR (95% CI)	I^2 heterogeneity statistic †
**2 weeks**	Unexposed	43676418	1007	Ref		Ref	
	Exposed	118598	13	6.12 (2.81 to 13.29)	48%, p-value 0.15	5.50 (2.82 to 10.71)	30%, p-value 0.24
**4 weeks**	Unexposed	43559417	1001	Ref		Ref	
	Exposed	235599	19	4.61 (2.17 to 9.78)	61%, p-value 0.08	4.36 (2.23 to 8.55)	51%, p-value 0.13
**6 weeks**	Unexposed	43442563	998	Ref		Ref	
	Exposed	352453	22	3.75 (1.60 to 8.77)	74%, p-value 0.02	3.66 (1.68 to 7.97)	68%, p-value 0.04
**Any time after 22 gestational weeks**	Unexposed	43257803	990	Ref		Ref	
	Exposed	537213	30	2.79 (1.18 to 6.64)	81%, p-value 0.01	2.37 (1.16 to 4.85)	71%, p-value 0.03

* Adjusted for maternal age at start of pregnancy, parity, education, income, living with a partner, region of birth, underlying chronic conditions and vaccination against SARS-CoV-2.

† The I^2 heterogeneity statistic and corresponding p-value for differences in the estimates across the three countries.

eTable 5 Risk of stillbirth with maternal SARS-CoV-2 infection after 22 completed gestational weeks sensitivity analysis excluding those who had tested positive any time prior to 22 competed gestational weeks

**Exposure window**	**SARS-CoV-2 infection**	**Follow-up time in days**	**Number of events**	**Unadjusted**		**Adjusted** [*]	
				HR (95% CI)	I^2 heterogeneity statistic [†]	HR (95% CI)	I^2 heterogeneity statistic [†]
**2 weeks**	Unexposed	42134055	974	Ref		Ref	
	Exposed	113048	14	6.74 (3.25 to 13.98)	44%, p-value 0.17	5.69 (3.18 to 10.16)	14%, p-value 0.31
**4 weeks**	Unexposed	42036204	969	Ref		Ref	
	Exposed	210899	19	5.30 (2.35 to 11.92)	67%, p-value 0.05	4.57 (2.33 to 8.99)	51%, p-value 0.13
**6 weeks**	Unexposed	41950543	966	Ref		Ref	
	Exposed	296560	22	4.52 (1.83 to 11.16)	77%, p-value 0.01	3.91 (1.81 to 8.42)	68%, p-value 0.05
**Any time after 22 gestational weeks**	Unexposed	41709379	957	Ref		Ref	
	Exposed	537724	31	2.82 (1.23 to 6.47)	80%, p-value 0.01	2.41 (1.22 to 4.77)	69%, p-value 0.04

[*] Adjusted for maternal age at start of pregnancy, parity, education, income, living with a partner, region of birth, underlying chronic conditions and vaccination against SARS-CoV-2.

[†] The I^2 heterogeneity statistic and corresponding p-value for differences in the estimates across the three countries.

References

1. Ludvigsson JF. The first eight months of Sweden's COVID-19 strategy and the key actions and actors that were involved. *Acta paediatrica (Oslo, Norway : 1992)* 2020;109:2459-71. doi: 10.1111/apa.15582
2. Ludvigsson JF. How Sweden approached the COVID-19 pandemic: Summary and commentary on the National Commission Inquiry. *Acta paediatrica (Oslo, Norway : 1992)* 2023;112:19-33. doi: 10.1111/apa.16535
3. Stephansson O, Pasternak B, Ahlberg M, et al. SARS-CoV-2 and pregnancy outcomes under universal and non-universal testing in Sweden: register-based nationwide cohort study. *BJOG : an international journal of obstetrics and gynaecology* 2021 doi: 10.1111/1471-0528.16990
4. The Public Health Agency of Sweden. Recommendations regarding COVID-19. Web page in Swedish [cited 2023 April 3]. Available from: https://www.folkhalsomyndigheten.se/smittskydd-beredskap/utbrott/aktuella-utbrott/covid-19/skydda-dig-sjalv-och-andra-rekommendationer-om-covid-19/.
5. Norwegian Institute of Public Health. Norwegian Surveillance System for Communicable Diseases (MSIS) 2022 Available from: https://www.fhi.no/en/hn/health-registries/msis/ accessed 20/01/2023.
6. Norwegian Health Directorate. How to increase the test capacity for SARS-CoV-2 in Norway? 2020 Available from: https://www.helsedirektoratet.no/rapporter/hvordan-oke-testkapasiteten-for-sars-cov-2-i-norge/Hvordan%20%C3%B8ke%20testkapasiteten%20for%20SARS-CoV-2%20i%20Norge.pdf/_/attachment/inline/5225947a-299b-4dbf-921f-a54fd74d1615:c8f6d0508b75e9b226284ec7cff3c76edc856e10/Hvordan%20%C3%B8ke%20testkapasiteten%20for%20SARS-CoV-2%20i%20Norge.pdf accessed 04.04.2023.
7. Voldstedlund M, Haarh M, Mølbak K. The Danish Microbiology Database (MiBa) 2010 to 2013. *Euro Surveill* 2014;19 doi: 10.2807/1560-7917.es2014.19.1.20667
8. Aabakke AJM, Krebs L, Petersen TG, et al. SARS-CoV-2 infection in pregnancy in Denmark-characteristics and outcomes after confirmed infection in pregnancy: A nationwide, prospective, population-based cohort study. *Acta Obstet Gynecol Scand* 2021;100:2097-110. doi: 10.1111/aogs.14252
